# Application of negative pressure to the periocular microenvironment to lower IOP in patients implanted with telemetric IOP sensor: report of two early studies of the FSYX ocular pressure adjusting pump

**DOI:** 10.3389/fopht.2026.1887910

**Published:** 2026-07-27

**Authors:** Michael D. Greenwood, Tanner J. Ferguson, Lars Choritz, Thomas W. Samuelson, Jamie J. Beckman, H. Burkhard Dick, Iqbal K. Ahmed, John P. Berdahl

**Affiliations:** 1 Vance Thompson Vision, Sioux Falls, SD, United States; 2 University Eye Clinic, Magdeburg, Germany; 3 Minnesota Eye Consultants, Minneapolis, MN, United States; 4 EyeCare Partners, Dearborn, MI, United States; 5 Ruhr University Bochum, University Eye Clinic Bochum, Knappschaft Kliniken, Bochum, Germany; 6 Alan S. Crandall Center for Glaucoma Innovation, University of Utah, Salt Lake City, UT, United States

**Keywords:** eyemate, glaucoma, intraocular pressure (IOP), normal-tension glaucoma (NTG), ocular pressure adjusting pump (OPAP)

## Abstract

**Introduction:**

The purpose of this study was to determine the effect on intraocular pressure (IOP) of applying negative pressure (NP) to the periocular space, as measured by an intraocular IOP sensor in human eyes.

**Methods:**

Eyes with open-angle glaucoma (OAG) and previously implanted with the eyemate^®^-IO IOP-measuring device (Implandata Ophthalmic Products GmbH (now G-Metrics GmbH)) wore an early prototype of the FSYX™ Ocular Pressure Adjusting Pump (OPAP; Balance Ophthalmics, Inc.) in two separate studies: “Eyemate 1” and “Eyemate 2.” In Eyemate 1, study eyes received periorbital NP application at -10mmHg with OPAP for 70 to 110 minutes vs no NP in the fellow control eye. In Eyemate 2, eyes wore OPAP for 8 hours of continuous NP application set to 50% of the eye’s baseline IOP vs no NP in the fellow control eye. In both studies, IOP was measured in real time using the implanted eyemate-IO device. The primary effectiveness endpoint in both studies was the change from baseline IOP in the study eye, as measured with eyemate-IO.

**Results:**

All seven study eyes demonstrated immediate and sustained reductions in IOP during NP application. In Eyemate 1, three study eyes had individual mean steady-state IOP reductions of 31%, 51%, and 23% from baseline. In Eyemate 2, four study eyes had a mean 8-hour IOP reduction of 20% from baseline. All patients tolerated goggle wear and NP application.

**Conclusion:**

These studies provided early proof-of-concept evidence that NP application to the periocular space lowers IOP, as measured by an implanted intraocular sensor, and was well tolerated over short-term use. Given the small, uncontrolled or open-label designs, these findings are hypothesis-generating; larger, controlled studies are required to confirm efficacy and characterize safety.

## Introduction

Glaucoma is the leading cause of irreversible blindness worldwide (1). Currently, the mainstay of treatment options targets the lowering of intraocular pressure (IOP), the only modifiable risk factor associated with disease progression (2). Current treatment options to lower IOP include medications, laser procedures, and surgeries (3–6). The most common treatment for glaucoma is with topical ocular medication, which lowers IOP effectively but has relatively low adherence rates and numerous side effects (7). Laser treatments and surgeries are also effective but can be difficult to manage, have high failure rates, and undesired complications (3).

This report presents early human data using a device to lower external atmospheric pressure around the periocular region to reduce IOP. These studies were conducted early in the clinical development program of the FSYX™ Ocular Pressure Adjusting Pump (OPAP; Balance Ophthalmics, Inc.), which was subsequently cleared by the United States Food and Drug Administration (FDA). The studies reported here were performed during the device’s early development, several years before that authorization, and are presented as proof-of-concept data. OPAP is a Class II device (a moderate-risk classification subject to FDA general and special controls) indicated for lowering IOP in patients with open-angle glaucoma (OAG) and daytime IOP ≤ 21 mmHg (8). The OPAP system comprises a pair of pressure-sensing goggles and a corresponding pressure-modulating pump, which are mechanically and pneumatically connected. The system includes a tubing system that permits independent NP application and subsequent IOP control for each eye. OPAP reduces IOP by decreasing atmospheric pressure around the eye (9). The mechanism of IOP reduction is based on the principles of hydrostatic physics, as described by Pascal’s law, and was previously described at length in a prior report, in addition to being cleared by the FDA (8, 9). Briefly, the OPAP goggles create a customizable low-pressure microenvironment around the orbital rim. Per Pascal’s law, the fluid pressure within the eye shifts toward equilibrium with the reduced external pressure, producing a measurable decrease in IOP. The IOP-lowering effect of OPAP (including its predecessor, the Multi-Pressure Dial) has been characterized in several prior studies of the device (10–14), and longer-term safety and effectiveness have been evaluated in the EXPLORER (15) and HERCULES (16) trials.

The present study evaluates OPAP’s IOP-lowering capability in eyes with a previously implanted intraocular wireless IOP sensor (eyemate^®^-IO, Implandata Ophthalmic Products GmbH, now G-Metrics GmbH). Prior studies have described the design and performance of the wireless, ring-shaped sensor, which is designed to be implanted in the ciliary sulcus (17). Adverse events associated with the intraocular configuration prompted the development of a suprachoroidally placed variant, for which dedicated safety and performance data have been reported (18, 19). The sensor permits high-frequency IOP monitoring without disturbing the eye (20), an appealing feature given the increased interest in IOP dynamics as they relate to glaucoma, with recent telemetric studies further characterizing short- and long-term IOP variability in primary open-angle glaucoma (21–25). Using an implanted sensor to measure IOP changes in response to negative periocular pressure provides an ideal opportunity test this proof of concept.

## Materials and methods

### Design

Inclusion criteria in both studies were broad and included any adult (age ≥ 18 years) with ≥ 1 eye implanted with eyemate device. Patients were ineligible if they had any of the following in either eye: macular degeneration, untreated retinal detachment, eyelid edema, or conjunctival chemosis. IOP measurements in both studies were collected via the wireless implanted IOP sensor eyemate-IO, which is a small pressure-sensing component designed to be placed in the ciliary sulcus during cataract surgery (26). The IOP sensor communicates with a handheld reader unit that conveys the IOP measurement values and also powers the sensor when placed within a specified distance. The wireless IOP sensor demonstrates favorable agreement with Goldmann applanation tonometry (GAT) measurements, supporting the reliability and performance of the device as an IOP measurement tool (27). Specifically, ARGOS-02 reported an intraclass correlation coefficient of approximately 0.78 between the eyemate-IO and GAT, with a systematic offset of approximately 3.2 mmHg. Because the sensor is housed within the ciliary sulcus and measures intraocular pressure directly, the periocular negative pressures applied in these studies were not expected to materially deform the sensor itself; however, potential effects of periocular pressure on the eye-sensor interface have not been formally quantified. All participants underwent a baseline ophthalmological examination and OPAP goggle fitting to ensure participant safety and a proper seal of the goggles prior to NP application. All participants provided written informed consent before any study-specific procedures were performed. Neither study was prospectively registered in a public clinical-trials registry; both were early proof-of-concept investigations, and the regulatory and ethics basis for each is detailed in the Ethics/Ethical Approval section. Because this was an early proof-of-concept study, no formal sample-size calculations were performed. The protocol did not require documentation of the duration of eyemate-IO.

Eyemate 1 was a single-site, single-visit, open-label, non-controlled study in which patients wore an OPAP prototype for a 25-minute ramp-down period, followed by either a 20- or 60-minute steady-state period, and concluded with a 25-minute ramp-up period. Participants could choose 20 or 60 minutes for the steady-state period. Following secure placement of the goggles, the pressure-modulating pump gradually applied NP at a rate of -2 mmHg every 5 minutes until the -10 mmHg target value was achieved within the goggles. The initial sequence was referred to as the “ramp-down” period. Following the ramp-down period, the intra-goggle negative pressure was sustained at -10 mmHg, referred to as the “steady-state” period. The final period was the “ramp-up” period, during which NP was incrementally released at the same rate as the ramp-down period (i.e., intra-goggle pressure increased by +2 mmHg every 5 minutes until the pressure within the goggles returned to 0 mmHg, matching ambient atmospheric pressure). The ramp-down and ramp-up methodology was a strategy employed in early OPAP studies for participant comfort; it is not required in clinical use, per the FDA-approved Instructions for Use. Eyemate 1 was first-in-human study of OPAP (Mercury Pressure Dial, at the time) did not include pre-specified safety endpoints; this is acknowledged as a limitation, and formal safety endpoints were added in subsequent studies as knowledge of OPAP’s safety profile evolved. The choice of differing negative-pressure magnitudes and steady-state durations between Eyemate 1 and Eyemate 2, and the participant-selected steady-state duration within Eyemate 1, reflect sequential exploration of the device’s operating envelope during early development rather than a pre-planned head-to-head comparison. With seven total participants, these studies were not powered to discriminate between settings and were designed as exploratory proof-of-concept.

IOP measurements were collected with the telemetric IOP sensor at baseline and then every five minutes until NP returned to 0 mmHg. At each 5-minute interval, three pressure values were collected, and the mean was recorded. If any of the three measurements differed by ≥1.0 mmHg, a fourth measurement was obtained and the mean of the three most similar measurements was recorded.

Eyemate 2 was a single-site, open-label, controlled study with three clinic visits. Visit 1 included informed consent, screening, and baseline assessments. During visit 2, participants wore the OPAP prototype for 8 hours, during which the study eye received consistent NP application at 50% of its baseline IOP, with applied negative pressure capped so as not to exceed -20 mmHg, while the control eye received no NP. In the study eye, thirteen IOP measurements were recorded with the implanted eyemate-IO sensor: one at baseline and four at each of hours 1, 4, and 7 (one every 15 minutes), sampling the early, middle, and late portions of the 8-hour wear period. Because the fellow (control) eye did not carry an implanted sensor, its IOP was assessed by pneumatonometry at the screening, day-0, and follow-up visits rather than continuously during NP application; outcome assessment was not masked. NP application ended after the 13th measurement (i.e., after 8 hours of exposure). Study Visit 3 was a safety follow-up visit that included a second ophthalmological exam between 5-to-8 days after the 8-hour NP application.

### Investigational device

**Figure 1:**
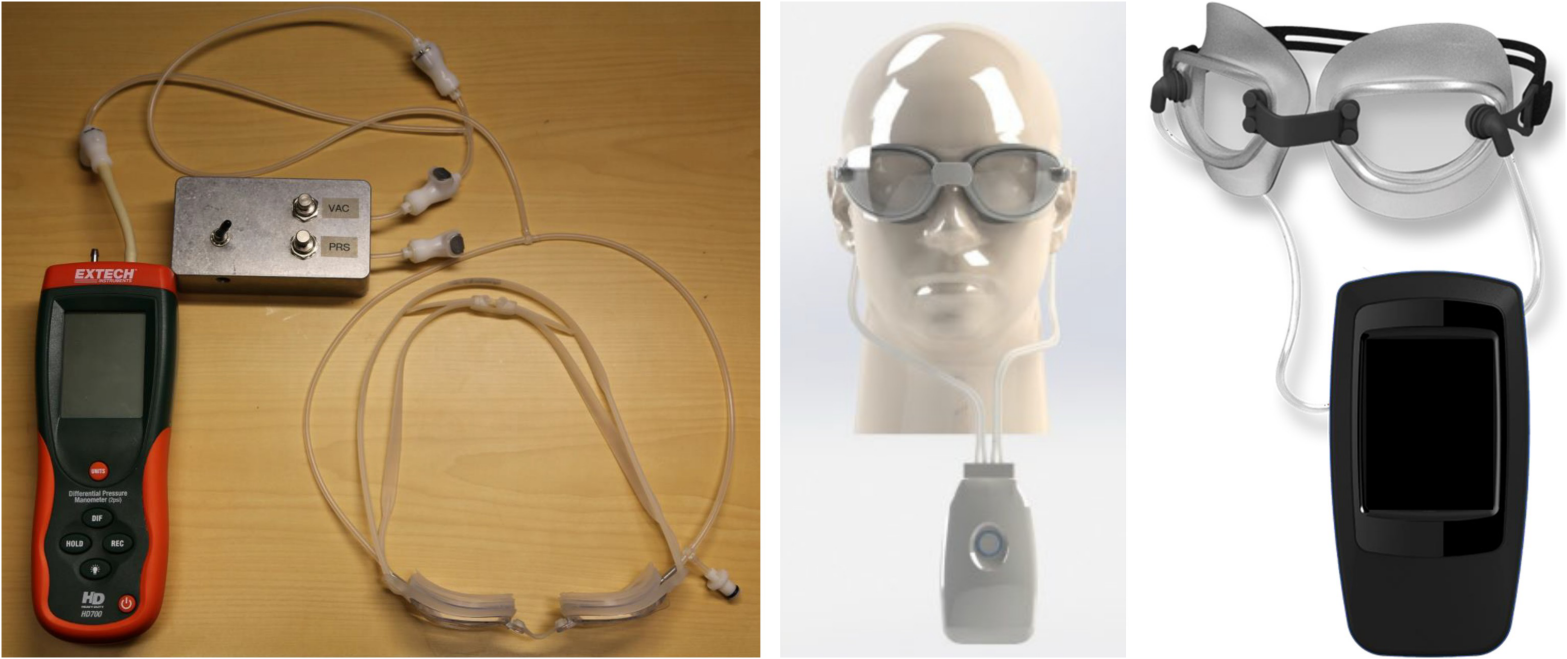
Prototypes used in Eyemate 1 (left) and Eyemate 2 (center) and the commercial, FDA-approved (right) FSYX Ocular Pressure Adjusting Pump. OPAP prototypes all included three key components: goggles, differential pressure manometer, and adjustable pump. All three components were connected to deliver negative pressure to the periocular region. FDA, United States Food and Drug Administration; OPAP, FSYX Ocular Pressure Adjusting Pump.

The OPAP systems used in these studies, shown in **Figure 1**, were prototypes of the current commercially available product and consisted of three key components: a pair of pressure-sensing goggles, a pressure-adjusting pump, and a commercial manometer. The goggles were connected to the pump and the differential pressure manometer via tubing to create a negative pressure microenvironment in each periorbital region.

### Statistical analysis

In Eyemate 1, IOP measurements were summarized with mean and standard deviation. Given the small sample size, no comparative statistical analyses were performed; results are reported descriptively as the mean steady-state IOP and the percentage change from baseline for each eye.

In Eyemate 2, continuous variables were summarized using descriptive statistics (sample size; mean; standard deviation; median; minimum and maximum). Discrete variables were summarized using frequencies and percentages. Adverse events were summarized by presenting the number and percentage of participants with any adverse event. Any other information collected, such as severity or relationship to study device, were listed. Because continuous implanted-sensor data were available only for the study eye, the primary analyses were within-eye paired comparisons of the study eye against its own baseline. For IOP measurements, paired-sample t-tests were employed to compare baseline IOP measurement to the IOP measurements obtained over the 8-hour period. Paired-sample t-tests were also conducted to compare the baseline IOP with recovery after negative pressure was discontinued. A two-sided p-value < 0.05 was considered statistically significant. Given the small sample (N = 4), these analyses are exploratory and were not powered to detect small treatment effects or rare adverse events.

## Results

### Eyemate 1

**Figure 2:**
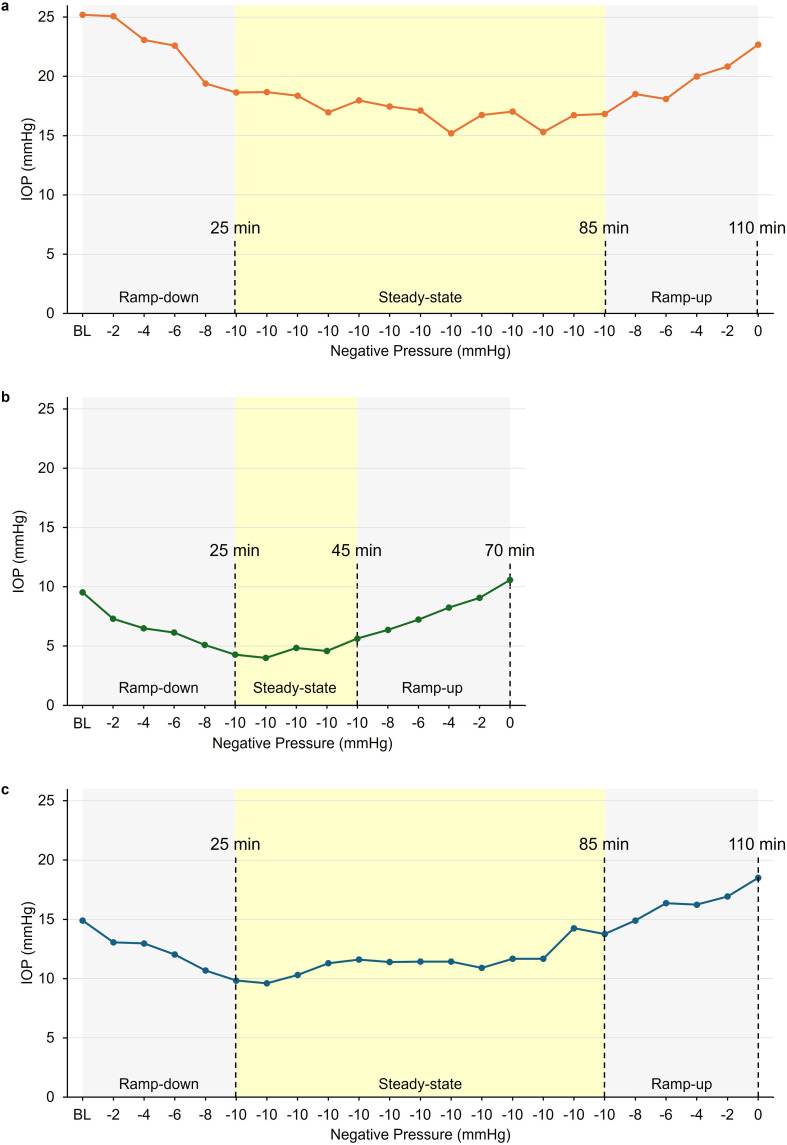
Individual IOP values over time in Eyemate 1. Negative pressure values along the x-axes presented in 5-minute increments, per study protocol. Panels **(a–c)** represent IOP over time for participants 1, 2, and 3, respectively. BL, baseline; IOP, intraocular pressure.

All three eyes (three patients) in Eyemate 1 had open-angle glaucoma and a previously implanted eyemate-IO sensor. Individual baseline IOPs ranged from 9.5 to 25.2 mmHg; two patients selected the 60-minute steady-state period and one selected the 20-minute period. IOP measurements during ramp-down, steady-state, and ramp-up are summarized in **Table 1** and shown over time at each measurement in **Figures 2a–c** for patients 1, 2, and 3, respectively. All three eyes demonstrated an immediate and sustained reduction from baseline during NP application with IOP returning to pre-study values after NP application stopped. At the end of the ramp-up period (no NP applied), measured IOP returned to baseline levels.

**Table 1:** IOP measurements in Eyemate 1.

IOP measurement, mmHg ± SD:	Patient 1	Patient 2	Patient 3
Visit 1: baseline	25.2 ± 1.0	9.5 ± 1.1	13.2 ± 0.1
Visit 2: NP application			
End of ramp-down (initial -10 mmHg NP)	18.6 ± 1.1	4.3 ± 0.2	9.8 ± 0.9
Midpoint of steady-state^a^	17.1 ± 1.0	4.8 ± 0.3	11.4 ± 0.6
End of steady-state	16.8 ± 0.7	5.6 ± 0.3	13.8 ± 0.6
End of ramp-up (no NP applied)	22.7 ± 1.5	10.5 ± 1.1	18.5 ± 1.0
Mean steady-state reduction, % (mmHg ± SD)	31% (-8.0 ± 1.1)	51% (-4.9 ± 0.7)	23% (-3.4 ± 1.6)

a. Because patients 1 and 3 chose the 60-minute steady-state, while patient 2 chose the 20-minute steady-state, the midpoint occurred after 30 minutes of -10 mmHg NP for patients 1 and 3, and after 10 minutes of -10 mmHg for patient 2. IOP, intraocular pressure; NP, negative pressure; SD, standard deviation.

- Patient 1 (60-minute steady-state): Across the 60-minute steady-state period, IOP decreased by an average of 31% (-8.0 ± 1.1 mmHg) from a baseline of 25.2 ± 1.1 mmHg.
- Patient 2 (20-minute steady-state): Across the 20-minute steady-state, IOP decreased by an average of 51% (-4.9 ± 0.7 mmHg) from a baseline of 9.5 ± 1.1 mmHg.
- Patient 3 (60-minute steady-state): Across the 60-minute steady-state, IOP decreased by an average of 23% (-3.4 ± 1.6 mmHg) from a baseline of 13.2 ± 0.1 mmHg.

### Eyemate 2

**Figure 3:**
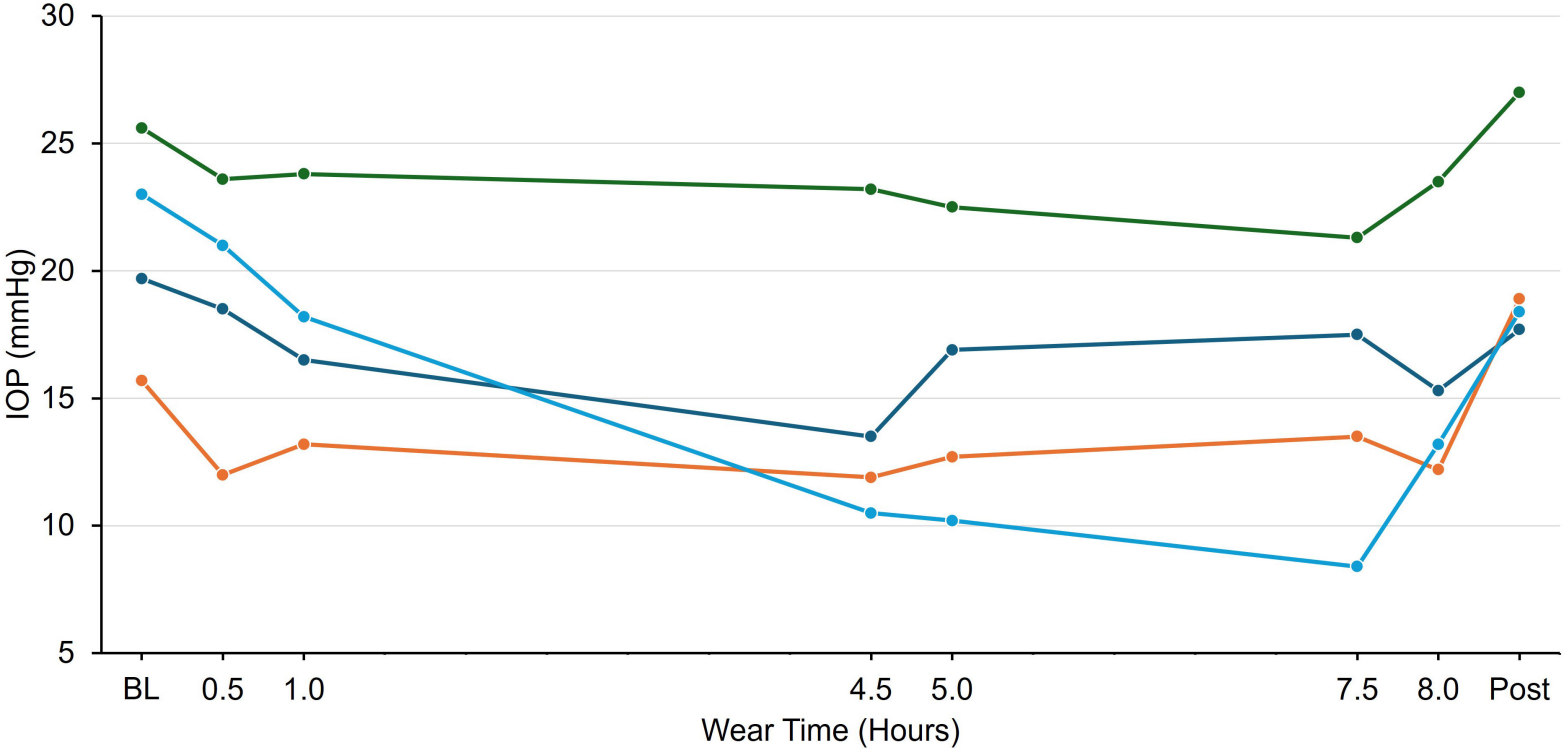
Individual IOP values over time in Eyemate 2. Negative pressure was applied consistently at 50% of the eye’s baseline IOP. Markers indicate IOP measurements for each eye at intervals specified in the study protocol. Lines represent individual eyes. BL, baseline; IOP, intraocular pressure.

**Figure 4:**
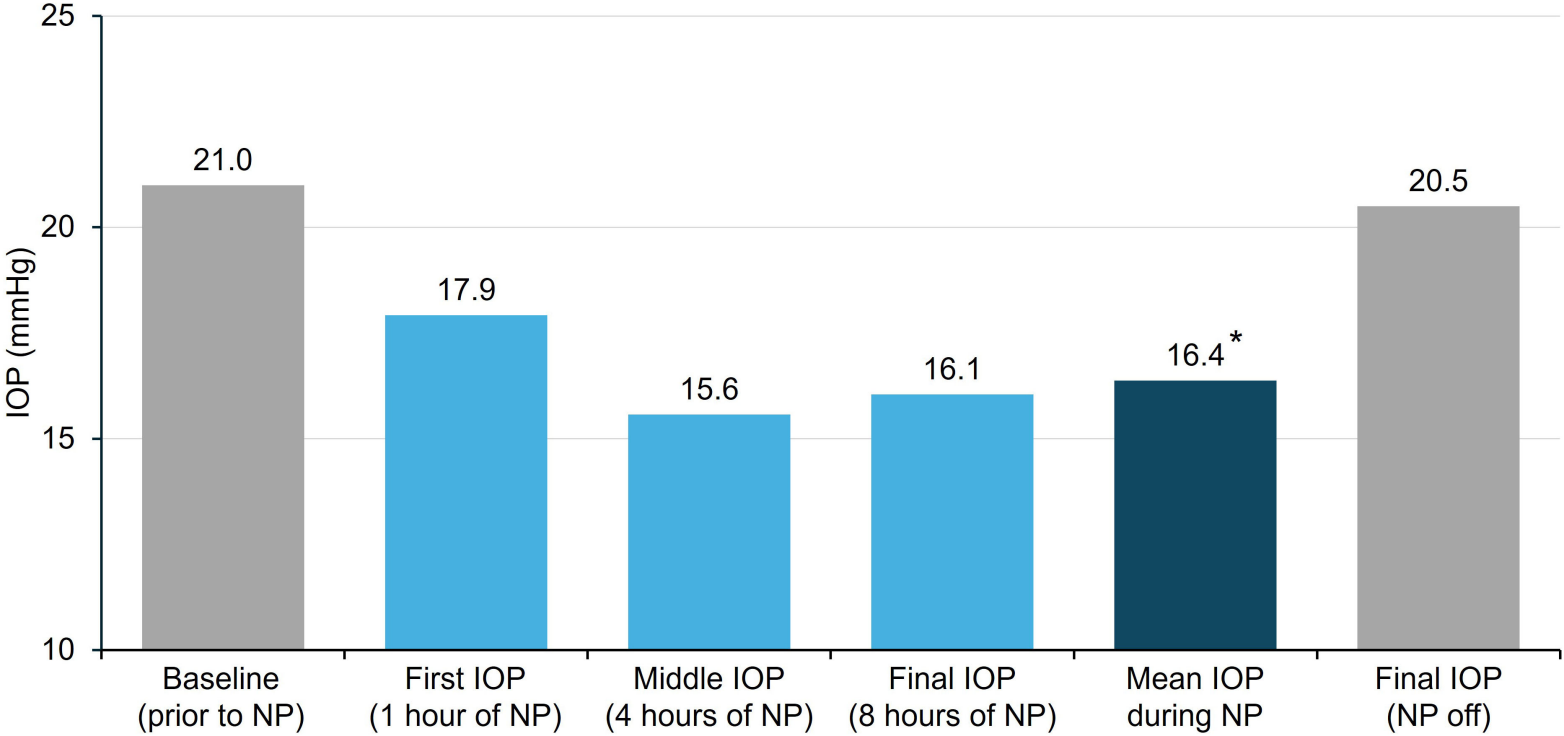
Mean IOP measurements in Eyemate 2. N = 4 eyes. Dark-blue column (second from right) represents mean IOP across all readings during NP application (light-blue columns). * Statistically significant (p < 0.05) reduction from baseline using 2-sided t-test. IOP, intraocular pressure; NP, negative pressure.

Six participants provided consent and were enrolled. One participant failed screening because of pre-existing herpes keratitis, and one was excluded from analysis because the implanted sensor produced IOP values that were inconsistent with simultaneous pneumatonometry (discrepancy > 15 mmHg on both the screening and study days). Four eyes (one eye from each of four patients) completed the study and were included in the analysis. IOP measurements for each eye receiving NP are shown in **Figure 3**. Mean IOP measurements for all eyes receiving NP at each timepoint are shown in **Figure 4**.

The four analyzed Eyemate 2 participants were 60 to 76 years of age (three male, one female), all white, and all with OAG and a previously implanted eyemate-IO sensor. Individual baseline (pre-NP) IOPs measured by the implanted sensor were 19.7, 15.7, 25.6, and 23.0 mmHg (mean 21.0 mmHg). Individual values at each measured timepoint are provided in **Table 2**.

**Table 2:** Individual IOP values in Eyemate 2 (mmHg), measured by the implanted eyemate-IO sensor.

Study eye	Baseline (pre-NP)	Mean during NP	Change from baseline	Immediately after NP
1	19.7	16.4	-17%	17.7
2	15.7	12.6	-20%	18.9
3	25.6	23.0	-10%	27.0
4	23.0	13.6	-41%	18.4
**Mean**	**21.0**	**16.4**	**-22%**	**20.5**

Values are means of the IOP measurements obtained at the protocol-specified timepoints. NP, negative pressure.

Means for each value shown in bold.

Safety was assessed in Eyemate 2 by slit-lamp examination, manifest refraction, best-corrected distance visual acuity, optic nerve head assessment (optical coherence tomography of the retinal nerve fiber layer and optic nerve head), visual field testing (24–2), and adverse-event monitoring at baseline, immediately after the 8-hour wear period, and at the 1-week follow-up visit. No serious adverse events occurred, and no clinically significant change from baseline was observed in slit-lamp findings, refraction, visual acuity, optical coherence tomography, or visual fields. Eleven adverse events were recorded across the enrolled participants, all graded as mild: lid edema or erythema, conjunctival chemosis or swelling (three events), ocular itching, mild non-ocular headache (two events), mild eye pain, a transient rise in IOP, and a skin lesion. All device-related ocular events were transient and resolved spontaneously without sequelae.

As shown in **Figure 4**, mean IOP before NP application was 21.0 ± 4.3 mmHg. During hour 1 of NP applied at 50% of baseline, mean IOP was 17.9 ± 4.4 mmHg. During hour 4 of NP applied at 50% of baseline, the mean IOP was 15.6 ± 5.4 mmHg. Mean IOP over the entire 8-hour period was 16.4 ± 4.9 mmHg. Mean IOP immediately after NP application was 20.5 ± 4.4 mmHg. NP application for 8 hours yielded a statistically significant treatment effect (p < 0.05) for OPAP, as measured by the within-eye reduction of IOP from baseline.

The mean IOP during hour 4 (15.6 mmHg) was lower than during hour 1 (17.9 mmHg), consistent with a sustained or progressive effect over the wear period rather than tachyphylaxis. Immediately after NP cessation, mean IOP (20.5 mmHg) returned to a value that did not differ significantly from baseline (21.0 mmHg; p = 0.06), indicating prompt reversibility without a statistically significant rebound above baseline.

## Discussion

These observations from early in the OPAP clinical program provide important proof of concept in establishing that NP application to the periocular space can reduce IOP in an immediate and reversible manner. The two studies used different negative-pressure settings (a fixed -10 mmHg in Eyemate 1 and 50% of baseline IOP in Eyemate 2), which is consistent with titratability; however, a within-patient dose-response was not formally tested. To the authors’ knowledge, this report describes the first in-human application of NP over the orbit to lower IOP measured by an implantable IOP sensor.

Beyond demonstrating the technical feasibility of using an implanted telemetric IOP sensor in this setting, the present work adds to the existing OPAP literature in two ways. First, it independently validates the titratability of OPAP across a range of starting IOPs, with reproducible IOP reductions observed at both a fixed NP setting (-10 mmHg) and a proportional titration (50% of baseline IOP). Second, it provides an additional measurement modality (implanted IOP sensor) alongside direct manometry and transcorneal (excursion) tonometry used in subsequent OPAP studies (28), which confirms that periocular NP produces a measurable, real-time reduction in IOP.

The wireless sensor is among several devices (29, 30) recently introduced to address the unmet need for 24-hour IOP monitoring. Because the sensor is implanted in the sulcus and measures absolute pressure, it circumvents the known issues with available tonometers and the impact of corneal biomechanics (31). However, the sensor has been shown to have a relatively wide margin of error, as compared to Goldmann applanation tonometry, that may exceed ±3 mmHg (18). This limitation was well understood going into the studies and was at least partially addressed by the aggregating multiple IOP measurements at each timepoint. As a proof-of-concept, the intention was to look for a pattern of repeatable IOP reduction with periocular NP applications, rather than prove any hypotheses. By design, these early studies were not powered to inform clinical decision-making. However, subsequent studies (10–12, 15) have thoroughly established the device’s safety profile, including favorable findings for nightly use for up to 3 months in the EXPLORER Trial (13) and up to a year in the pivotal HERCULES Trial (16).

In all seven patients in this report, OPAP immediately lowered IOP upon NP application, and IOP returned to baseline levels upon restoring the periocular pressure to ambient atmospheric pressure. The findings observed in this report also highlight OPAP’s versatility. Individual baseline IOPs in Eyemate 1 ranged from 9.5 to 25.2 mmHg. The application of -10 mmHg effectively lowered IOP in all these patients, even when their baseline IOP was < 10 mmHg. In one patient, IOP remained < 6 mmHg (4.7 ± 0.7 mmHg) throughout the steady-state period with no adverse events; this transient, asymptomatic ocular hypotony resolved after cessation of NP with no sequelae or other adverse events. OPAP’s ability to lower IOP in patients with lower baseline IOP (e.g., normal-tension glaucoma) evolved into one of the device’s core strengths during its clinical development and led to approval as the first device to address nocturnal IOP elevations in patients with controlled daytime IOP (i.e., ≤ 21 mmHg).

The relationship between OPAP and ocular perfusion pressure (OPP) warrants brief discussion. OPP is commonly approximated as the difference between mean arterial pressure (MAP) and IOP (OPP ≈ MAP − IOP). Because OPAP lowers IOP without altering systemic blood pressure, the expected effect on OPP is a net increase. Low OPP has been associated with glaucomatous optic neuropathy progression (32–34). Therefore, an IOP-lowering intervention that does not depress MAP is mechanistically attractive in glaucoma management.

Individual responses to NP varied within these small cohorts, including one Eyemate 1 eye in which IOP fell to approximately 4 mmHg during the steady-state phase. Despite this transient hypotony, no deleterious effects on the optic nerve, retinal nerve fiber layer, or visual function were observed in the prospective safety assessment performed in Eyemate 2. Subsequent, larger trials, including the pivotal HERCULES Study, have demonstrated consistent IOP-lowering, with the most common adverse event being transient eyelid edema and no signal of optic nerve or visual-field deterioration attributable to NP application or to the periodic IOP fluctuations it produces.

While all participants in the present studies had OAG, specific subclassification by tension category (e.g., high-tension vs. normal-tension) or disease stage was not stratified in these proof-of-concept analyses given the small samples. Notably, the IOP-lowering effect of OPAP in eyes with relatively low baseline IOP (directly relevant to normal-tension glaucoma) was subsequently the focus of dedicated studies including HERCULES, which examined OPAP in patients with controlled daytime IOP and demonstrated clinically meaningful nocturnal IOP reduction with OPAP (16).

These studies are limited mainly due to small sample sizes, and by additional design factors: Eyemate 1 was uncontrolled and Eyemate 2 was open-label, with the untreated fellow eye assessed only at discrete visits rather than by continuous implanted-sensor monitoring, so a concurrent quantitative control was not available; outcome assessment was unmasked; both studies were underpowered to detect small effects or rare adverse events; the steady-state duration in Eyemate 1 was participant-selected; baseline IOP was measured on a single occasion, so diurnal variation was not accounted for; the 50%-of-baseline titration target used in Eyemate 2 is not a standard approach; potential contralateral or systemic effects of unilateral NP were not assessed; and neither study was prospectively registered. However, considering that the availability of patients implanted with the eyemate is limited, and this is a proof of concept for NP application using an implanted IOP sensor, these studies provide novel and important findings. The titratable and reliable action of OPAP observed in these studies helped inform larger subsequent evaluations and commercialization. This was a small case series with only seven patients. Future studies with larger sample sizes and longer wear times were subsequently performed (11–16).

## Conclusions

These early studies provided proof-of-concept evidence that applying NP to the periocular space lowers IOP in an immediate and reversible manner, as measured by an implantable IOP sensor. To the authors’ knowledge, this is the first such demonstration using an implanted IOP sensor. This was found to be particularly useful in treating nocturnal IOP elevations. In summary, the Eyemate Studies demonstrated a proof of concept for OPAP’s mechanical mechanism of action, supporting the hypothesis that NP application with OPAP produces an IOP-lowering effect that is immediate, titratable, and reversible, even in patients with a low baseline IOP.

## Data Availability

The raw data supporting the conclusions of this article will be made available by the authors, upon reasonable request, without undue reservation.
